# GLP-1 medications: use and interest in a representative survey of Finns

**DOI:** 10.1038/s41366-026-02149-5

**Published:** 2026-07-07

**Authors:** Piia Tuuli Jallinoja, Kirsi H. Pietiläinen, Virginia W. Chang

**Affiliations:** 1 Health Sciences Unit, Faculty of Social Sciences, Tampere University, Tampere, Finland; 2 Obesity Research Unit, Research Program for Clinical Metabolism, Faculty of Medicine, University of Helsinki and Healthy Weight Hub, Abdominal Center, Helsinki University Hospital and University of Helsinki, Helsinki, Finland; 3 Department of Social and Behavioral Sciences, NYU School of Global Public Health and Department of Population Health, NYU Grossman School of Medicine, New York University, New York, USA

**Keywords:** Weight management, Preventive medicine

## Abstract

**Background and objectives:**

GLP-1–based medications have rapidly reshaped the landscape of obesity treatment. Semaglutide was approved for obesity treatment in 2021 in the US and 2022 in Europe, sparking global interest, and the GLP-1/GIP dual-agonist tirzepatide has demonstrated even greater efficacy. However, survey-based data on who uses or considers these medications—particularly across socioeconomic groups, BMI categories, and weight management experiences—remain limited.

**Subjects/methods:**

A nationally representative online survey of Finnish adults (*n* = 1729, of which 1693 were included) was conducted in June 2025 via a market research company using quota sampling. Use and awareness of GLP-1 medications for obesity were measured with a single item listing widely known brands—Ozempic, Wegovy, Zepbound, and Mounjaro—to aid recognition. In regression analyses, current, past, and potential users (*n* = 322) were combined.

**Results:**

In total, 3.5% reported current and 2.0% past use, and 13.5% expressed interest in future use. Notably, ~40% of individuals with obesity reported no interest. Bivariate analysis showed that current use was more common among women, those aged 50–69, those with household income exceeding €70,000, individuals with higher BMI, frequent weight loss attempts, experiences of weight-based discrimination, and self-blame. Multivariable analysis showed that current, past, and potential use had strongest associations with BMI ≥30.0 kg/m², repeated or persistent weight loss attempts, experiences of discriminatory treatment due to weight, self-blaming thoughts and hopeful perceptions of GLP-1 medications. Concern about serious health risks was associated with lower likelihood of use or interest.

**Conclusions:**

GLP-1–based medications are gaining recognition, but uptake remains heterogeneous and many individuals with obesity remain uninterested. Concerns about potential health risks persist. Attitudes toward these medications are shaped not only by weight status but also by prior weight management experiences and perceptions. Clinical communication should be sensitive to these factors.

## Introduction

Semaglutide (2.4 mg) received regulatory approval for the treatment of obesity in the United States in 2021 [1], and Europe in 2022 [2], following randomized controlled trials demonstrating substantial weight reduction in individuals with overweight or obesity, regardless of type 2 diabetes status [3, 4]. Semaglutide is a glucagon-like peptide-1 (GLP-1) receptor agonist that enhances satiety, suppresses hunger, reduces energy intake, and improves glycemic control by stimulating glucose-dependent insulin secretion, suppressing glucagon release, and delaying gastric emptying [5]. Tirzepatide, a dual agonist targeting GLP-1 as well as GIP (gastric inhibitory peptide) receptors, has since demonstrated even greater efficacy in promoting weight loss relative to GLP-1 monotherapy [6, 7].

Since 2021, Ozempic—a brand name for semaglutide—has become a widely recognized shorthand for this class of medications in public and media discourse [8]. Formally, Ozempic (semaglutide 1 mg) is approved for type 2 diabetes, while Wegovy (semaglutide 2.4 mg) is indicated for obesity [2]. However, widespread off-label use of Ozempic as a substitute for Wegovy has blurred these distinctions in public discourse. Tirzepatide is approved in Europe under the brand name Mounjaro for the treatment of both type 2 diabetes and obesity [9], while in the United States, Mounjaro is indicated for diabetes, and Zepbound for obesity [10, 11]. Liraglutide, an earlier GLP-1 receptor agonist, is also approved for both indications (Victoza for diabetes and Saxenda for obesity) [12, 13], but its clinical use has declined with the emergence of longer-acting and more efficacious agents [14, 15].

With conventional lifestyle counselling, sustained weight loss has been difficult to achieve. The arrival of the newest GLP-1 medications has altered this landscape to such an extent that, in popular and media discourse, these therapies are often portrayed as transformative “miracle drugs” promising not only weight loss but also personal reinvention [8, 16]. Accordingly, demand has surged in several countries [15, 17–19], while traditional behavioural programmes, such Weight Watchers, have seen declining engagement and revenue [20]. Despite the enthusiasm, concerns have been raised. The most common adverse effects include nausea, vomiting, diarrhea, constipation, and abdominal discomfort, with rarer reports of pancreatitis, gallbladder disease, and dehydration [3, 7, 10, 21]. Additional risks include muscle loss, malnutrition in certain populations, and potential misuse, especially among persons with eating disorders [22, 23]. Broader concerns relate to the longer-term economic impact of these drugs for individuals as well as public healthcare systems and concerns around health equity.

Despite the growing clinical relevance, little is known about the actual users and interest in using these medications in real-world settings. While data from clinical trials provide insight into efficacy and safety, they do not capture broader population-level patterns of use. Survey-based data on who uses or seeks these medications—particularly across different BMI categories, socioeconomic backgrounds, and prior weight management experiences—remain scarce. To date, only a few population surveys on perceptions of GLP-1 medications have been published, such as one in Saudi Arabia [24], two in the United States [25, 26], one in the United Kingdom [27] and one in Jordania [28]. Of these, only the US study uses data that are weighed to be representative of adult population. Another US study targeted a university student population [29].

These earlier studies have shown that interest in using GLP-1 medications was associated with higher BMI, greater weight concerns, more body shame, greater maladaptive weight loss behaviours (such as skipping meals), and lower body appreciation [29]. Awareness of GLP-1 medications was higher among women than men, among those who had tried to lose weight compared to those who had not [27] and among higher income groups [26]. Finally, current medication users trusted the effectiveness and safety of these medications more than non-users [27].

There is a notable lack of research based on representative population samples that examines individuals’ experiences with GLP-1 medications and their associations with sociodemographic and weight-related factors. This study addressed this gap by utilizing representative, population-based data. First, we conduct bivariate analysis to explore the associations between GLP-1 medication use (categorized as current use, past use, potential use, lack of interest, and lack of awareness) and key variables including gender, age, education, income, BMI, weight-related experiences and perceptions of GLP-1 medications. These analyses are performed in two directions: (1) assessing the distribution of GLP-1 use and awareness across sociodemographic and weight-related factors, and (2) examining the sociodemographic and weight-related profiles within each GLP-1 user category. In the final phase, multivarible regression is employed to identify the associations of sociodemographic, weight-related and medication-related factors with a combined outcome variable representing current, past, or potential use of GLP-1 medications.

This study contributes novel data to inform clinical practice, public health guidance, and responsible messaging around weight loss pharmacotherapy.

### The study setting

Finland, the setting of the study, offers a Nordic welfare state context for GLP-1 medication use. The health care system is based on universal access, with publicly funded services available to all residents [30]. While private and employer-based services exist, the system emphasizes equity, affordability, and preventive care.

Over 60% of adults have overweight or obesity (BMI ≥ 25) and over 26% have obesity (BMI ≥ 30) in 2023 [31], placing Finland at the higher end of the European spectrum [32]. Since 2021, the use of GLP-1 medications has steadily increased [15], with prescriptions issued through obesity care units, public healthcare centres, and private clinics. Monthly costs range from 100€ to 550€ depending on dosage and reimbursement. Without reimbursement (e.g., for type 2 diabetes), the price can be a substantial barrier for low-income Finns. Regarding reimbursement policy, liraglutide 3 mg (Saxenda) and naltrexone-bupropion (Mysimba) are currently eligible for restricted basic reimbursement for obesity, while liraglutide 1.8 mg (Victoza) and semaglutide (Ozempic, Rybelsus) are reimbursed only for treatment of type 2 diabetes. Wegovy and Mounjaro are not reimbursed for obesity treatment. In 2025, semaglutide was by far the most commonly prescribed medication for obesity [15].

There is potential in Finland for both acceptance and scepticism regarding GLP-1 medications. As a high-trust society, Finland is characterized by strong confidence in physicians [33] and scientific expertise [34, 35]. Trust in pharmaceuticals compared to ‘natural remedies’ increased until 2019, but has somewhat decreased since then [35].

In summary, population characteristics such as high prevalence of obesity and trust in medical solutions may support the uptake of GLP-1 medications, while a portion of the population may remain hesitant, or unable to access these treatments due to financial or other barriers.

## Methods

### Data

A survey was conducted as part of Obesity Stigma Project funded by the Ministry of Social Affairs and Health, Finland. The survey was commissioned from Taloustutkimus Oy, which conducted the data collection between June 16 and June 25, 2025, among members of its consumer panel living in mainland Finland. Taloustutkimus, a leading market research company in Finland, uses quota sampling to ensure representativeness in terms of age, gender, and region. Younger age groups are slightly oversampled to account for typically lower response rates. During data collection, quotas are actively monitored, and reminders are sent as needed to maintain balanced participation.

The questionnaire covered topics such as respondents’ weight management history, current weight and height, use of GLP-1 medications, beliefs about the causes and responsibilities for obesity, and views on various obesity management approaches, including GLP-1 medications.

### Measures

For this study, an item was included that assessed both use and awareness of newly available medications used for weight loss. The question was intentionally framed using the term ‘obesity medications’ to prompt respondents to consider their use and interest specifically in the context of obesity, rather than type 2 diabetes. This terminology also aligns with language commonly used in the popular press, whereas medical terms like semaglutide and GLP-1 receptor agonists may have been less familiar to the respondents. To aid recognition, the questionnaire listed brand names—Ozempic, Wegovy, Zepbound, and Mounjaro—as examples of products that respondents may have encountered through national or international media coverage. Although only Wegovy and Monjaro currently hold official indications for obesity treatment in Europe, in practice Ozempic is frequently prescribed in Finland for individuals with obesity alone, without other comorbid conditions. The respondents were asked “Have you heard about the new obesity medications, such as Ozempic, Wegovy, Zepbound, and Mounjaro?” with answering options: “Yes, and I currently use myself”, “Yes, and I have earlier used (don’t use anymore)”, “Yes, and I am interested in using”, “Yes, but I have not used myself, nor am I interested in using” and “I have not heard of these medications”. Although the questionnaire did not use the term “GLP-1 medications”, we refer to this five-category outcome as the “GLP-1 use” variable for brevity. In the limitations, we discuss limitation of this question. Unless otherwise indicated, all variables in the study are operationalized as categorical variables.

We included the following sociodemographic variables: gender, age, household yearly gross income, and education. We refer to gender as the respondents self-defined identification as it was asked in the questionnaire, with three answering options (“man”, “woman”, “other”). BMI was calculated from self-reported weight and height using the formula: weight (in kg) divided by height squared (m^2^). Table 2 displays the categories used in our analyses.

Past weight loss attempts were assessed with the question: “Have you ever tried to lose weight during your life?” (“No”, “No, but I try to keep my weight steady”, “Yes, 1–2 times during my life”, “Yes, 3 times or more during my life”, “Yes, I am constantly trying to lose weight”). Weight-related experiences and thoughts were ascertained with the following questions: “Have you been treated unfairly or discriminated against because of your weight during the past year” (“Never”, “Seldom”, “Sometimes”, “Often”, “I don’t know”), and “I blame myself for my weight” (“Fully disagree”, “Somewhat disagree”, “Neither agree, not disagree”, “Somewhat agree”, “Fully agree”, “I don’t know”).

GLP-1 medication-related perceptions were assessed with items preceded by the header “In recent years, pharmaceutical companies have developed new medications, such as Ozempic, Wegovy, Mounjaro and the like. In the following, we ask for your views on them”. The following statements were included in the analysis: “I am hopeful that the obesity medications will help people effectively lose weight” and “I am concerned that serious health risks may emerge from new medications” (“Fully disagree”, “Somewhat disagree”, “Neither agree nor disagree”, “Somewhat agree”; “Fully agree”, “I don’t know”).

The study was conducted in accordance with the guidelines by the Finnish National Board on Research Integrity (TENK). The study does not fall under the scope of Finnish Medical Research Act and Decree, and it does not include particularly sensitive questions. Therefore, Tampere University does not mandate an ethics board review for this type of research. To ensure compliance, the legal advisor of Tampere Region Research Ethics Board was consulted, who confirmed that an ethical review was not required and was unnecessary for confirming the study’s ethical soundness.

All participants were recruited voluntarily through Taloustutkimus Oy, a reputable research company, ensuring informed participation. The original data held by Taloustutkimus are pseudonymized, as the company retains the ability to identify respondents. However, the data provided to the research team were anonymized.

### Statistical analysis

Respondents who identified as other than female or male were excluded from the analysis due to their small number (*n* = 11, 0.6%). Additionally, 24 respondents were dropped due to missing data on weight or height and one respondent due to unreliable height and weight responses, resulting in a final analytic sample of 1693 participants. To maintain full comparability between the bivariate and multivariable analyses, we used a consistent analytic sample and applied the same exclusions in all models.

First, sociodemographic characteristics of the sample are presented alongside population data from Statistics Finland for comparison.

Second, we conduct bivariate analyses for associations of the GLP-1 use variable with sociodemographic variables, BMI category, and the various other weight- and medication-related variables. Statistical significance was assessed with Pearson’s chi-square when its assumptions were met (≤20% of expected cell counts < 5 and none < 1); otherwise, exact p-values were calculated using the Fisher–Freeman–Halton test with Monte Carlo estimation (two-sided).

These results are presented in two tables, one displaying row percentages, and another presenting column percentages, as we felt that each was informative.

Table 3 showing column percentages additionally reports mean age and BMI values across the five GLP-1 use categories, with ANOVA used for statistical testing.

Third, we examine the association of GLP-1 use with the various covariates in a multivariable context. Due to relatively small numbers for current and former use, the response categories “I am currently using,” “I have used in the past,” and “I am interested in using” were combined into a single category and modelled as a dichotomous variable representing current, past, or potential use (*n* = 322, 19.0%). Logistic regression was then used to model the association of this composite variable with covariates. Model 1 includes sociodemographic factors and BMI as independent variables, while Model 2 adds variables capturing past weight loss attempts, weight-related unfair treatment, self-blame, and hopeful and concerned perceptions of weight loss medications.

Categories were also merged for other variables due to low frequencies. For the item on experiences of discrimination and unfair treatment, the categories of “never” and “don’t know” were combined, as were “sometimes” and “often”. Similarly, in the items measuring self-blame, and perceptions of GLP-1 medications, the responses “fully agree” and “somewhat agree” were combined, while the remaining categories were grouped together. SPSS version 29.0 was used for all analyses.

## Results

Table 1 presents characteristics of the survey respondents. A comparison with Statistics Finland’s reports [36] indicates that the sample is representative in terms of gender, age and geographical distribution. Individuals with only elementary level education were underrepresented. Compared with the nationally representative Healthy Finland Study 2024, the present sample shows a slight overrepresentation of individuals with obesity (29.6% vs. 24.5%) [37, 38].

**Table 1:** Sample sociodemographic characteristics and comparison to Statistics Finland’s reports * (*n* = 1693).

	Survey	Population data
		Statistics Finland 31.12.2024*
**Gender**		Aged 18 years and older
Men	49.0	49.2
Women	51.0	50.8
**Age**		Youngest age group: 20–29 years
18–29 years	15.8	14.5
30–49 years	32.1	33.0
50–69 years	30.2	30.6
70 years or older	21.9	21.9
**Region**		Aged 18 years and older
Uusimaa region, including the capital area	31.4	31.5
Southern Finland (other than Uusimaa region)	22.3	20.9
Western Finland	24.3	25.0
Eastern and Northern Finland	22.0	22.6
**Education**		Aged 15 years and older
Elementary/comprehensive school	13.9	24.9
Vocational school	35.3	39.7 (vocational school and high school together)
High school	14.2	
Post-secondary vocational school	7.7	22.7 (Post-secondary vocational school, bachelor’s degrees and similar together)
Bachelor’s degree from university of applied sciences	8.5	
Master’s degree from university of applied sciences	2.4	
Bachelor’s degree from university	4.7	
Master’s degree from university	12.5	11.5
Doctoral degree	0.8	1.2

*Statistics Finland. Population structure and educational structure of population, https://stat.fi/en 2025.

Table 2 shows bivariate associations of the GLP-1 use variable with sociodemographic variables, BMI category, and other weight- and medication-related variables. A majority of respondents (74.3%) had heard of these medications. Current use was reported by 3.5% of participants, while 2.0% indicated past use. 13.5% expressed interest in using these medications.

**Table 2:** Sample characteristics by GLP-1 use categories, row frequencies (%) (*N* = 1693).

		Current user	Former user	Interested in using	Not interested in using	Has not heard		
	*n* (%)	%	%	%	%	%	Total	*P* value
TOTAL	1693	3.5	2.0	13.5	55.3	25.7	100.0	
**GENDER**								
Men	830 (49.0)	2.9	1.8	10.1	53.0	32.2	100.0	
Women	863 (51.0)	4.2	2.2	16.7	57.5	19.5	100.0	*p* < 0.001*
**AGE**								
18–29 yrs	268 (15.8)	1.5	0.4	9.7	65.3	23.1	100.0	
30–49 yrs	543 (32.1)	3.2	1.3	16.2	62.3	17.0	100.0	
50–69 yrs	512 (30.2)	6.2	3.9	16.6	45.9	27.6	100.0	
70+ yrs	370 (21.9)	1.6	1.6	8.3	50.3	38.2	100.0	*p* < 0.001*
**EDUCATION**								
Elementary/Comprehensive school	236 (13.9)	4.6	2.5	11.3	46.3	35.4	100.0	
High school/ Vocational school	838 (49.5)	3.5	2.2	15.2	53.7	25.3	100.0	
Bachelor’s degree/ Univ. of applied sciences	394 (23.3)	3.7	2.2	14.4	56.2	23.4	100.0	
Master’s degree/PhD	225 (13.3)	1.7	0.0	8.7	68.1	21.4	100.0	*p* < 0.001*
**HOUSEHOLD YEARLY GROSS INCOME**								
Under 20,000 €	333 (19.7)	2.1	0.3	12.9	61.9	22.8	100.0	
20,001–40,000 €	409 (24.2)	3.9	3.7	13.7	50.6	28.1	100.0	
40,001–70,000 €	414 (24.5)	3.6	2.4	15.5	53.6	24.9	100.0	
70,001 € +	302 (17.8)	6.3	1.3	14.9	55.3	22.2	100.0	
I do not want to answer	235 (13.9)	1.3	1.7	8.5	57.0	31.5	100.0	*p* < 0.001*
**BMI (kg/m²)**								
Underweight ( < 18.50)	34 (2.0)	0.0	3.0	0.0	54.5	42.4	100.0	
Normal weight (18.50–24.99)	550 (32.5)	0.5	0.9	3.8	66.1	28.6	100.0	
Overweight (25.00–29.99)	609 (36.0)	2.0	0.8	10.7	57.0	29.5	100.0	
Obesity I (30.00–34.99)	277 (16.4)	7.6	3.2	25.6	41.9	21.7	100.0	
Obesity II (35.00–39.99)	133 (7.9)	7.5	5.3	30.8	42.9	13.5	100.0	
Obesity III (≥40.00)	90 (5.3)	15.6	7.8	32.2	37.8	6.7	100.0	*p* < 0.001 #
**PAST WEIGHT LOSS ATTEMPTS DURING LIFE**								
No	174 (10.3)	0.6	0.0	1.7	49.4	48.3	100.0	
No, but I try to keep my weight steady	345 (20.4)	0.3	0.3	3.8	60.0	35.7	100.0	
1–2 times	387 (22.9)	0.5	1.0	9.6	63.8	25.1	100.0	
3+ times	442 (26.1)	5.4	2.5	17.9	58.8	15.4	100.0	
Yes, constantly	345 (20.4)	9.3	5.2	27.8	39.4	18.3	100.0	*p* < 0.001*
**TREATED UNFAIRLY OR DISCRIMINATED AGAINST BECAUSE OF THEIR WEIGHT DURING THE PAST YEAR**								
Never	1253 (74.0)	2.3	1.0	9.4	59.0	28.3	100.0	
Seldom	203 (12.0)	3.4	3.0	22.7	47.3	23.6	100.0	
Sometimes	153 (9.0)	9.8	7.2	28.8	43.8	10.5	100.0	
Often	31 (1.8)	19.4	6.5	22.6	32.3	19.4	100.0	
I do not know	53 (3.1)	5.7	5.7	24.5	45.3	18.9	100.0	*p* < 0.001 #
**“I BLAME MYSELF FOR MY WEIGHT”**								
Fully or somewhat agree	706 (41.7)	6.9	3.3	23.4	50.1	16.3	100.0	
Neither agree nor disagree	273 (16.1)	2.2	2.6	7.7	54.6	33.0	100.0	
Fully or somewhat disagree	690 (40.8)	0.7	0.6	5.8	61.2	31.7	100.0	
I do not know	24 (1.4)	0.0	0.0	8.3	45.8	45.8	100.0	*p* < 0.001*
**“I AM HOPEFUL THAT THE OBESITY MEDICATIONS WILL HELP PEOPLE EFFECTIVELY LOSE WEIGHT”**								
Fully or somewhat agree	824 (48.7)	5.8	2.5	23.9	49.6	18.1	100.0	
Neither agree nor disagree	322 (19.0)	1.9	2.5	4.7	63.4	27.6	100.0	
Fully or somewhat disagree	336 (19.8)	1.8	1.5	2.4	67.6	26.8	100.0	
I do not know	211 (12.5)	0.0	0.0	3.8	45.5	50.7	100.0	*p* < 0.001*
**“I AM CONCERNED THAT SERIOUS HEALTH RISKS MAY EMERGE FROM NEW MEDICATIONS”**								
Fully or somewhat agree	1157 (68.3)	2.6	2.1	10.8	60.3	24.2	100.0	
Neither agree nor disagree	224 (13.2)	4.5	1.8	18.8	47.3	27.7	100.0	
Fully or somewhat disagree	158 (9.3)	10.1	1.9	22.8	46.8	18.4	100.0	
I do not know	154 (9.1)	2.6	1.9	16.2	37.7	41.6	100.0	*p* < 0.001*

* p-value based on Pearson’s chi-square (assumptions met).

# p-value based on Fisher–Freeman–Halton exact test (Monte Carlo, 2-sided).

Women more often reported current use (4.2% vs. 2.9%) and interest in future use (16.7% vs. 10.1%), whereas men were more likely to be unaware of GLP-1 medications (32.2% vs. 19.5%). Individuals aged 50–69 were the most likely to be current users, while those aged 70+ were most often unaware of GLP-1 medications. Respondents with a master’s degree were least likely to report current or past use or express interest in future use. In terms of income, current use was most common among those with household incomes exceeding €70,000, while lack of interest was most pronounced in the lowest income group.

Current use and interest in GLP-1 medications increased with BMI (Table 2). While 3.8% of individuals with normal BMI expressed interest, 37.8% of respondents with BMI ≥ 40.0 kg/m² did so. However, a notable proportion of those in the groups with BMI ≥ 30.0 kg/m²—ranging from 37.8 to 42.9%—reported no interest in using these medications. Awareness also varied by BMI, increasing as BMI rose.

Current and past use as well as interest in using GLP-1 medications increased gradually with the frequency of past weight loss attempts (Table 2). Respondents who had sometimes or often experienced unfair treatment due to their weight in the past year were more likely to be current or past users than others. Respondents with self-blaming thoughts more often than others were current or past users, as well as interested in using GLP-1 medications. Those who were hopeful about the potential of GLP-1 medications were more frequently users than others. In contrast, those who hold concerns about the serious health risks, were more often not interested in using these medications.

Table 3 presents the cross tabulations of Table 2 with column, rather than row, percentages. Individuals aged 50–69 were notably overrepresented among both current and past users. Mean age was highest among former users (57.3 yrs) and those who had not heard of GLP-1 medications (56.7) and lowest among those not interested in using (49.3) and those interested (50.4). Regarding income, current users were most commonly found in the highest income bracket (over €70,000), whereas former users were more frequently from households with annual income below €40,000.

**Table 3:** Sample characteristics by GLP-1 use categories, column frequencies (%), means of age and BMI (*N* = 1693).

		Current user	Former user	Interested in using	Not interested in using	Has not heard	
N	1693	60	34	228	936	435	
	*n* (%)	%	%	%	%	%	*P* value
**GENDER**							
Men	830 (49.0)	40.0	44.1	36.8	47.0	61.4	
Women	863 (51.0)	60.0	55.9	63.2	53.0	38.6	
Total		100.0	100.0	100.0	100.0	100.0	*p* < 0.001*
**AGE**							
*Mean*	*All: 51.64*	*54.5*	*57.3*	*50.4*	*49.3*	*56.7*	*p* < *0.001 +*
18–29 yrs	268 (15.8)	6.7	2.9	11.4	18.7	14.3	
30–49 yrs	543 (32.1)	30.0	20.6	37.7	36.2	21.4	
50–69 yrs	512 (30.2)	53.3	58.8	37.3	25.2	32.0	
70+ yrs	370 (21.9)	10.0	17.6	13.6	19.9	32.4	
Total		100.0	100.0	100.0	100.0	100.0	*p* < 0.001*
**EDUCATION**							
Elementary/Comprehensive school	236 (13.9)	18.3	17.6	11.8	11.8	18.9	
High school/ Vocational school	838 (49.5)	50.0	55.9	54.8	48.2	49.0	
Bachelor’s degree/ Univ. of applied sciences	394 (23.3)	25.0	26.5	24.6	23.8	20.9	
Master’s degree/PhD	225 (13.3)	6.7	0.0	8.8	16.2	11.3	
Total		100.0	100.0	100.0	100.0	100.0	*p* < 0.001*
**HOUSEHOLD YEARLY GROSS INCOME**							
Under 20,000 €	333 (19.7)	11.7	2.9	18.9	22.0	17.5	
20,001–40,000 €	409 (24.2)	26.7	44.1	24.6	22.1	26.4	
40,001–70,000 €	414 (24.5)	25.0	29.4	28.1	23.7	23.7	
70,001 € +	302 (17.8)	31.7	11.8	19.7	17.8	15.4	
I do not want to answer	235 (13.9)	5.0	11.8	8.8	14.3	17.0	
Total		100.0	100.0	100.0	100.0	100.0	*p* < 0.001*
**BMI (kg/m²)**							
*Mean*	*All: 28.1*	*35.4*	*33.7*	*32.7*	*27.0*	*26.5*	*p* < *0.001 +*
Underweight (<18.50)	34 (2.0)	0.0	2.9	0.0	2.0	3.2	
Normal weight (18.50–24.99)	550 (32.5)	5.0	14.7	9.2	38.8	36.3	
Overweight (25.00–29.99)	609 (36.0)	20.0	14.7	28.5	37.2	41.1	
Obesity I (30.00–34.99)	277 (16.4)	35.0	26.5	31.6	12.3	13.8	
Obesity II (35.00–39.99)	133 (7.9)	16.7	20.6	18.0	6.1	4.1	
Obesity III (≥40.00)	90 (5.3)	23.3.	20.6	12.7	3.6	1.4	
Total		100.0	100.0	100.0	100.0	100.0	*p* < 0.001 #
**PAST WEIGHT LOSS ATTEMPTS DURING LIFE**							
No	174 (10.3)	1.7	0.0	1.3	9.2	19.3	
No, but I try to keep my weight steady	345 (20.4)	1.7	2.9	5.7	22.1	28.3	
1–2 times	387 (22.9)	3.3	11.8	16.2	26.4	22.3	
3+ times	442 (26.1)	40.0	32.4	34.6	27.8	15.6	
Yes, constantly	345 (20.4)	53.3	52.9	42.1	14.5	14.5	
Total		100.0	100.0	100.0	100.0	100.0	*p* < 0.001*
**TREATED UNFAIRLY OR DISCRIMINATED AGAINST BECAUSE OF THEIR WEIGHT DURING THE PAST YEAR**							
Never	1253 (74.0)	48.3	35.3	51.8	79.0	81.6	
Seldom	203 (12.0)	11.7	17.6	20.2	10.3	11.0	
Sometimes	153 (9.0)	25.0	32.4	19.3	7.2	3.7	
Often	31 (1.8)	10.0	5.9	3.1	1.1	1.4	
I do not know	53 (3.1)	5.0	8.8	5.7	2.6	2.3	
Total		100.0	100.0	100.0	100.0	100.0	*p* < 0.001 #
**“I BLAME MYSELF FOR MY WEIGHT”**							
Fully or somewhat agree	706 (41.7)	81.7	67.6	72.4	37.8	26.4	
Neither agree nor disagree	273 (16.1)	10.0	20.6	9.2	15.9	20.7	
Fully or somewhat disagree	690 (40.8)	8.3	11.8	17.5	45.1	50.3	
I do not know	24 (1.4)	0.0	0.0	0.9	1.2	2.5	
Total		100.0	100.0	100.0	100.0	100.0	*p* < 0.001*
**“I AM HOPEFUL THAT THE OBESITY MEDICATIONS WILL HELP PEOPLE EFFECTIVELY LOSE WEIGHT”**							
Fully or somewhat agree	824 (48.7)	80.0	61.8	86.4	43.7	34.3	
Neither agree nor disagree	322 (19.0)	10.0	23.5	6.6	21.8	20.5	
Fully or somewhat disagree	336 (19.8)	10.0	14.7	3.5	24.3	20.7	
I do not know	211 (12.5)	0.0	0.0	3.5	10.3	24.6	
Total		100.0	100.0	100.0	100.0	100.0	*p* < 0.001*
**“I AM CONCERNED THAT SERIOUS HEALTH RISKS MAY EMERGE FROM NEW MEDICATIONS”**							
Fully or somewhat agree	1157 (68.3)	50.0	70.6	54.8	74.6	64.4	
Neither agree nor disagree	224 (13.2)	16.7	11.8	18.4	11.3	14.3	
Fully or somewhat disagree	158 (9.3)	26.7	8.8	15.8	7.9	6.7	
I do not know	154 (9.1)	6.7	8.8	11.0	6.2	14.7	
Total		100.0	100.0	100.0	100.0	10.0.0	*p* < 0.001*

* p-value based on Pearson’s chi-square (assumptions met).

# p-value based on Fisher–Freeman–Halton exact test (Monte Carlo, 2-sided) due to >20% of expected cell counts < 5.

Mean BMI was highest among users (35.4 kg/m²) and former users (33.7 kg/m²) and lowest among those not interested in using (27.0 kg/m²) and those who had not heard of GLP-1 medications (26.5 kg/m²) (Table 3). Among users, 75.0% had a BMI ≥ 30.0 kg/m², 53.3% reported constant weight loss efforts and 40.0% having attempted to lose weight at least three times during life, 46.7% reported having faced weight-related unfair treatment or discrimination in the past year and 81.7% blamed themselves for their weight. Respondents who were either not interested in or unaware of GLP-1 medications tended to have lower BMI classifications, most commonly falling within the normal or overweight range. They were also more likely to report no history of weight loss attempts, no experiences of weight-based discrimination or unfair treatment in the past year and expressed lower levels of self-blame related to their weight.

Among current users and interested individuals, optimism about the effectiveness of GLP-1 medications was high (80.0% and 86.4% respectively), though about half also expressed concerns about serious health risks (Table 3). In contrast, those not interested or unaware were more likely to express concern than hope.

Finally, Table 4 displays the multivariable logistic regression using the composite GLP-1 use outcome, which combines current, past, or potential use. For brevity, we will refer to this as “use” in this section. Model 1 includes sociodemographics and BMI as independent variables. Women, individuals with household income over €40 000, and individuals with a BMI ≥ 25.00 kg/m² had significantly higher odds of use relative to the reference categories. For BMI, the odds ratios increased in a monotonic fashion above the normal weight reference group.

**Table 4:** Associations of GLP-1 medication use (current, past, or potential) with sociodemographic factors, BMI (kg/m²) and other weight-related factors, logistic regression, odds ratios (OR) and 95% confidence intervals (C.I.) (*n* = 1693).

	*n*	Model 1		Model 2	
		OR	95% C.I.	OR	95% C.I.
**GENDER**					
Men	830	1		1	
Women	863	1.84***	1.40–2.43	1.27	0.92–1.77
**AGE**					
18–29 yrs	268	1		1	
30–49 yrs	543	0.90	0.55–1.49	0.96	0.55–1.67
50–69 yrs	512	1.29	0.79–2.09	1.83*	1.06–3.17
70+ yrs	370	0.60	0.35–1.05	0.83	0.44–1.55
**EDUCATION**					
Elementary/comprehensive school	236	1		1	
High shool/vocational school	838	0.90	0.60–1.37	0.92	0.57–1.48
Bachelor’s degree/ Univ. of applied sciences	394	1.01	0.63–1.62	1.06	0.62–1.82
Master’s degree / PhD	225	0.65	0.35–1.20	0.58	0.29–1.15
**HOUSEHOLD YEARLY GROSS INCOME**					
Under 20,000 €	333	1		1	
20,001–40,000 €	409	1.52	0.99–2.34	1.34	0.82–2.20
40,001–70,000 €	414	1.66*	1.08–2.56	1.44	0.88–2.37
70,001 € +	302	1.71*	1.07–2.73	1.58	0.92–2.73
I do not want to answer	235	0.77	0.45–1.33	0.76	0.40–1.42
**BMI (kg/m²)**					
Underweight (<18.50)	34	0.61	0.08–4.68	0.26	0.31–2.23
Normal weight (18.50–24.99)	550	1		1	
Overweight (25.00–29.99)	609	2.84***	1.81–4.47	1.75*	1.05–2.91
Obesity I (30.00–34.99)	277	9.89***	6.23–15.70	3.89***	2.27–6.66
Obesity II (35.00–39.99)	133	12.84***	7.61–21.68	4.43***	2.36–8.35
Obesity III (≥40.00)	90	19.83***	11.08–35.50	4.90***	2.40–10.01
**PAST WEIGHT LOSS ATTEMPTS DURING LIFE**					
No/No, but I try to keep my weight steady	519			1	
1–2 times	387			1.74	0.93–3.25
3 + times	442			3.30***	1.84–5.90
Yes, constantly	345			6.14***	3.38–11.19
**TREATED UNFAIRLY OR DISCRIMINATED AGAINST BECAUSE OF THEIR WEIGHT DURING THE PAST YEAR**					
Never/Do not know	1306			1	
Seldom	203			1.65*	1.06–2.55
Sometimes/Often	184			3.64***	2.28–5.82
**“I BLAME MYSELF FOR MY WEIGHT”**					
Other answering options	987			1	
Fully/somewhat agree	706			2.07***	1.44–2.98
**“I AM HOPEFUL THAT THE OBESITY MEDICATIONS WILL HELP PEOPLE EFFECTIVELY LOSE WEIGHT”**					
Other answering options	869			1	
Fully/somewhat agree	824			6.27***	4.37–9.01
**“I AM CONCERNED THAT SERIOUS HEALTH RISKS MAY EMERGE FROM NEW MEDICATIONS”**					
Other answering options	536			1	
Fully/somewhat agree	1157			0.31***	0.22–0.43
Nagelkerke R Square		0.268		0.495	

* *p* < 0.05; ** *p* < 0.01; *** *p* < 0.001.

Model 2 adds the various other weight- and medication-related variables. Women continue to have higher odds of use, but the coefficient is greatly attenuated and no longer statistically significant. Those aged 50–69 years have 83% higher odds of use relative to those who 18–29 years old. For education, the highest category had lower odds of use relative to the lowest category, but this difference was not significant. While all income categories were estimated to have higher odds of use relative to the lowest category, these differences were not significant. BMI category remained a strong and significant predictor of use. Relative to the normal weight category, persons with overweight had 75% higher odds of use, persons with obesity 1 had 3.89 times the odds of use, and those with obesity 2 and obesity 3 categories had over 4 times the odds of use. Past weight loss attempts, unfair treatment/discrimination, and self-blame were also strongly and significantly associated with use, despite adjusting for BMI. Being hopeful about GLP-1 medications was associated with sixfold odds of use. Conversely, being concerned about serious health risks from GLP-1 medications was associated with 69% lower odds of use.

## Discussion

This study is among the first to examine the use of and interest in GLP-1 medications within a nationally representative adult sample. In the survey, GLP-1 medications were framed as ‘obesity medications’ to prompt respondents to consider their use in the context of obesity, rather than type 2 diabetes.

GLP-1 medications have attracted considerable attention in both national and international media [8, 16] highlighting their growing clinical relevance. This visibility has been amplified by widespread social media marketing and influencer engagement [16, 39]. As a result, by June 2025, 74.3% of Finnish adults were aware of GLP-1 medications. Among respondents, 3.5% reported current use, 2.0% past use, and 13.5% interest in future use, while 55.3% were not interested. Even among individuals with a BMI ≥ 30.0 kg/m², approximately 40% reported no interest. Notably, interest extended beyond BMI-based eligibility: 10.7% of respondents with overweight and 3.8% with normal BMI expressed interest. National purchase statistics report that 108 000 Finns ( ≈ 2,5% of adults) used weight loss medications in 2025 [15], broadly aligning with the proportion identified in our sample.

In bivariate analyses, women were more likely than men to be current users of GLP-1 medications and to express interest in their use. Similar association were reported in Finnish medication purchases statistics [15]. Awareness was also higher among women (80.5%) compared to men (67.8%), reflecting similar gender disparities reported in a UK study (87.2% vs. 68.2%) [27]. These patterns align with broader social norms in which weight control and dietary restraint are more salient among women [40, 41]. However, gender was not significant predictor in multivariable models for current, past, or potential use once weight loss and stigma-related factors were accounted for.

Interestingly, in bivariate analysis, former users were more commonly found in the €20,001–40,000 income bracket. This is notable given the out-of-pocket cost of GLP-1 medications, which may present financial barriers to initiating or maintaining use, particularly for individuals in lower and lower-middle income groups. In multivariable analyses income was generally positively associated with current, past, or potential use, but did not retain statistical significance in the full model.

In bivariate analyses, BMI, prior weight loss attempts, and other weight-related experiences were associated with use and awareness of the GLP-1 medications. Consistent with Markley et al. [29], use, as defined in the multivariable analysis of our study, was associated with higher BMI, repeated weight loss attempts, negative weight-related experiences, and self-blaming thoughts.

The association with BMI is not surprising. Association with prior weight loss efforts is understandable, too, as these earlier efforts may have reinforced the perception that sustainable weight loss is difficult, increasing openness to pharmacological solutions. Notably, negative weight-related experiences and self-blaming thoughts were associated with current, former, or potential use independently of BMI.

A notable share in every BMI category reported no interest in GLP-1 medications. These respondents showed greater concern about risks, lower expectations of effectiveness, and fewer experiences of weight-related discrimination, unfair treatment or self-blame. A UK study showed similar results [27]. While our sample size limits deeper subgroup analysis, this area warrants further investigation.

From a healthcare perspective, GLP-1 users and potential users often carry long histories of weight management shaped by stigma, societal judgment, and self-blame. For many, these medications represent both hope and uncertainty as they navigate new therapeutic options, potential adverse effects, and evolving understandings of weight loss. These findings underscore the need for communication that is sensitive to medical, financial, cultural and psychosocial realities of living with higher weight.

Several limitations should be noted. First, the lack of statistical significance for some estimates in our multivariable models may reflect insufficient power given the magnitude of the estimates. Second, the questionnaire did not capture participants’ health conditions, such as diabetes or eating disorders, which may influence engagement with GLP-1 medications. Third, because the survey listed only newer medications, earlier agents (e.g., Mysimba, Victoza, Saxenda) were not captured, potentially causing some misclassification of past use. However, national purchase data since 2019 indicate that these earlier medications account for only a small share relative to semaglutide in 2025 [15], making substantial underestimation of prior use unlikely. Fourth, the lack of data on the number of adults in each household limits the accuracy of household income estimates relative to household size. Fifth, individuals with only elementary level education were underrepresented.

In conclusion, although GLP-1 medications are becoming more widely recognized, many individuals with obesity remain uninterested, and concerns about health risks persist. These findings reflect the Finnish context—a Nordic welfare state high income levels [42] and strong public trust in medical and healthcare institutions [33]. Future research should compare countries with varying income structures, healthcare systems, and cultural attitudes toward medicine and pharmaceuticals, to examine how systemic contexts shape public perceptions and adoption of GLP-1s. Finally, in clinical practice, GLP-1 users and prospective users should not be approached solely through an obesity-focused lens. Their long histories of weight-management and experiences of stigma call for respectful, informed communication and care.

## Data Availability

The data used in this article (i.e., variables included in the reported analyses) are available from the author upon reasonable request.

## References

[1] FDA. Wegovy—semaglutide. U.S. Food and Drug Administration, 2021.

[2] EMA. Wegovy—semaglutide. European Medicines Agency, 2022.

[3] Wilding JPH, Batterham RL, Calanna S, Davies M, Van Gaal LF, Lingvay I, et al. Once-weekly semaglutide in adults with overweight or obesity. NEJM. 2021;384:989–1002. 10.1056/NEJMoa2032183 33567185

[4] Davies M, Færch L, Jeppesen OK, Pakseresht A, Pedersen SD, Perreault L, et al. Semaglutide 2· 4 mg once a week in adults with overweight or obesity, and type 2 diabetes (STEP 2): a randomised, double-blind, double-dummy, placebo-controlled, phase 3 trial. Lancet. 2021;397:971–84. 10.1016/S0140-6736(21)00213-0 33667417

[5] Nauck MA, Quast DR, Wefers J, Pfeiffer AF. The evolving story of incretins (GIP and GLP-1) in metabolic and cardiovascular disease: a pathophysiological update. Diab Obes Metab. 2021;23:5–29. 10.1111/dom.14496 34310013

[6] Aronne LJ, Horn DB, le Roux CW, Ho W, Falcon BL, Gomez Valderas E, et al. Tirzepatide as Compared with Semaglutide for the Treatment of Obesity. NEJM. 2025;393:26–36. 10.1056/NEJMoa2416394 40353578

[7] Jastreboff AM, le Roux CW, Stefanski A, Aronne LJ, Halpern B, Wharton S, et al. Tirzepatide for obesity treatment and diabetes prevention. NEJM. 2025;392:958–71. 10.1056/NEJMoa2410819 39536238

[8] Hari J Magic Pill: The Extraordinary Benefits and Disturbing Risks of the New Weight Loss Drugs. London: Bloomsbury Publishing; 2024.

[9] EMA. Mounjaro. European Medicines Agency, https://www.ema.europa.eu/en/medicines/human/EPAR/mounjaro, 2022.

[10] FDA. ZEPBOUND® (tirzepatide) Injection, for subcutaneous use. U.S. Food and Drug Administration, 2025.

[11] FDA. Mounjaro. U.S. Food and Drag Administration, https://www.accessdata.fda.gov/drugsatfda_docs/label/2022/215866s000lbl.pdf, 2022.

[12] EMA. Saxenda. European Medicines Agency, https://www.ema.europa.eu/en/medicines/human/EPAR/saxenda, 2025.

[13] EMA. Victoza. European Medicines Agency, https://www.ema.europa.eu/en/medicines/human/EPAR/victoza, 2009.

[14] Ukhanova, M Trends in glucagon-like peptide 1 receptor agonist prescribing patterns. The American Journal of Managed Care. 2025;1. 10.37765/ajmc.2025.89778 40829097

[15] Kela. Lihavuuslääkkeitä käyttää jo yli 100 000 suomalaista – kasvu jatkuu erittäin voimakkaana. The Social Insurance Institution of Finland. 2026. https://tietotarjotin.fi/uutinen/1361928/lihavuuslaakkeita-kayttaa-jo-yli-100-000-suomalaista-kasvu-jatkuu-erittain-voimakkaana?q=semaglutiini. Accessed 3 March 2026.

[16] Harjunen, H, Puhakka, A (Re) conceptualizing fatness as a medical and moral issue in the Finnish media discourse on fat and Ozempic in 2022–2023. Fat Studies. 2025.

[17] Mailhac A, Pedersen L, Pottegård A, Søndergaard J, Mogensen T, Sørensen H, et al. Semaglutide (Ozempic®) use in Denmark 2018 through 2023. Clin Epidemiol. 2024;16:307–18. 10.2147/CLEP.S456170 PMC11057509 38685990

[18] Yeo YH, Rezaie A, Hsieh TY-J, Hu X, Gaddam S, Ma KS-K, et al. Shifting trends in the indication of glucagon-like peptide-1 receptor agonist prescriptions: a nationwide analysis. Ann Intern Med. 2024;177:1289–91. 10.7326/M24-0019 39038291

[19] Ruiz PLD, Hindenes LB, Karlstad Ø, Nøkleby K, Meyer HE, Mailhac A, et al. Trends in the use of drugs with weight-loss effect: Scandinavian study from 2017 to 2023. Diab Obes Metab. 2025;27:2901–5. 10.1111/dom.16291 40017022

[20] Reuters. Weight Watchers files for chapter 11 bankruptcy to cut debt. 2025. https://www.reuters.com/business/weightwatchers-plans-file-bankruptcy-protection-2025-05-06/. Accessed 1 Nov 2025.

[21] FDA. WEGOVY (semaglutide) injection, for subcutaneous use, 2025.

[22] Bartel S, McElroy SL, Levangie D, Keshen A. Use of glucagon-like peptide-1 receptor agonists in eating disorder populations. Int J Eat Disord. 2024;57:286–93. 10.1002/eat.24109 38135891

[23] Mozaffarian D, Agarwal M, Aggarwal M, Alexander L, Apovian CM, Bindlish S, et al. Nutritional priorities to support GLP-1 therapy for obesity: a joint Advisory from the American College of Lifestyle Medicine. Obes Pillars. 2025;15:100181. 10.1016/j.obpill.2025.100181 PMC12264624 40673264

[24] Algarni MA, Algarni AAM, Alqarni WA, Alqassim AY. Knowledge and Attitude of the General Population in Saudi Arabia Toward Weight Management Medications (WMMs): A Cross-Sectional Study. Cureus. 2023;15:e42875. 10.7759/cureus.42875 PMC10474326 37664345

[25] Pew Research Centre. How Americans view weight-loss drugs and their potential impact on obesity in the U.S. www.pewcentre.com, 2024.

[26] KFF. KFF Health Tracking Poll May 2024: The Public’s Use and Views of GLP-1 Drugs. KFF, 2024.

[27] Auerbach N, Liu VN, Huang D, Clift AK, Al-Ammouri M, El-Osta A. What are community perspectives and experiences around GLP-1 receptor agonist medications for weight loss? A cross-sectional study in the UK. BMC Pub Health. 2025;3:e002519. 10.1136/bmjph-2024-002519 PMC12306312 40734969

[28] Abutaima R, Barakat M, Sawan HM, Al Omari SM, Mhaidat NM. Knowledge, attitudes, and practices towards the use of GLP-1 receptor agonists for weight loss among the general population in Jordan; A cross-sectional study. PloS one. 2024;19:e0314407. 10.1371/journal.pone.0314407 PMC11620468 39636914

[29] Markey CH, August KJ, Malik D, Richeson A. Body image and interest in GLP-1 weight loss medications. Body Image. 2025;53:101890. 10.1016/j.bodyim.2025.101890 40267815

[30] Tynkkynen L-K, Keskimäki I, Karanikolos M, Litvinova Y. Finland: health system summary 2023. European Observatory on Health Systems and Policies, 2023.

[31] Lehtoranta L, Kaartinen N, Jääskeläinen T, Mäki P, Pietiläinen K, Sares-Jäske L, et al. Lihavuus [Obesity]. Finnish Institute for Health and Welfare. 2023. https://www.thl.fi/tervesuomi_verkkoraportit/ilmioraportit_2023/lihavuus.html. Accessed 16 May 2026.

[32] EurostatOverweight and obesity - BMI statistics. Luxembourg: Publications Office of the European Union; 2025.

[33] Saarinen AO, Räsänen P, Kouvo A. Two dimensions of trust in physicians in OECD-countries. Int H Health Care Qual Assur. 2016;29:48–61. 10.1108/ijhcqa-04-2015-0041 27172620

[34] Wellcome Global Monitor. How does the world feel about science and health. London: Wellcome Trust; 2018. https://wellcome.org/reports/wellcome-global-monitor/2018.

[35] Tieteen tiedotus. Tiedebarometri [Science Barometer]. Helsinki: Tieteen tiedotus; 2024. https://www.tieteentiedotus.fi/files/Tiedebarometri_2024.pdf

[36] Statistics Finland. Population structure and educational structure of population. https://stat.fi/en/statistics, 2025.

[37] THL. Lihavien osuus - Terve Suomi 2024 [The prevalence of obesity - Healthy Finland Survey 2024]. Finnish Institute For Health and Welfare. 2025. https://www.thl.fi/tervesuomi_verkkoraportit/kyselyosuus_2024/ath_obesity_bmi30.html. Accessed 16 May 2026.

[38] THL. Quality report: The adult population’s well-being and health – Healthy Finland survey. Finnish Institute for Health and Welfare. 2025. https://thl.fi/en/data-and-statistics/data-quality/quality-reports-on-statistics/quality-report-the-adult-population-s-well-being-and-health-healthy-finland-survey#quality-report-the-adult-populations-well-being-and-health-healthy-finland-survey. Accessed 16 May 2026.

[39] Han SH, Safeek R, Ockerman K, Trieu N, Mars P, Klenke A, et al. Public interest in the off-label use of glucagon-like peptide 1 agonists (Ozempic) for cosmetic weight loss: a google trends analysis. Aesthet Sur J. 2024;44:60–7. 10.1093/asj/sjad211 37402640

[40] Brumberg JJ Fasting girls: The emergence of anorexia nervosa as a modern disease. New York: Vintage Books; 1988. 10.1126/science.240.4855.1061 17731731

[41] Harjunen H. Gendered fat bodies as neoliberal bodies. In: The contemporary reader of gender and fat studies. London & New York: Routledge; 2023. p. 30–40.

[42] Eurostat. Key figures on Europe. 2025 edition. Luxembourg: Publications Office of the European Union; 2025.

